# Surface guided radiation therapy: An international survey on current clinical practice

**DOI:** 10.1016/j.tipsro.2022.03.003

**Published:** 2022-03-30

**Authors:** V. Batista, M. Gober, F. Moura, A. Webster, M. Oellers, M. Ramtohul, M. Kügele, P. Freislederer, M. Buschmann, G. Anastasi, E. Steiner, H. Al-Hallaq, J. Lehmann

**Affiliations:** a Department of Radiation Oncology, Heidelberg University Hospital, Heidelberg, Germany; b Heidelberg Institute of Radiation Oncology (HIRO), National Center for Radiation Oncology (NCRO), Heidelberg, Germany; c Department of Radiation Oncology, Medical University of Vienna, Austria; d Institute for Radiation Oncology and Radiotherapy, Landesklinikum Wiener Neustadt, Austria; e Hospital CUF Descobertas, Department of Radiation Oncology, Lisbon, Portugal; f Radiotherapy and Proton Beam Therapy, University College Hospital, London, United Kingdom; g MAASTRO Clinic, Department of Medical Physics, Maastricht, the Netherlands; h Department of Medical Physics, Queen Elizabeth Hospital, University Hospitals Birmingham; i Department of Haematology, Oncology and Radiation Physics, Skåne University Hospital, Lund, Sweden; j Department of Radiation Oncology, University Hospital, LMU Munich, Munich, Germany; k St. Luke’s Cancer Centre, Royal Surrey Foundation Trust, Radiotherapy Physics, United Kingdom; l Department of Radiation and Cellular Oncology, University of Chicago, USA; m Radiation Oncology Department, Calvary Mater Newcastle, Australia; n School of Information and Physical Sciences, University of Newcastle, Callaghan, Australia; o Institute of Medical Physics, University of Sydney, Australia; p Department of Clinical Sciences, Medical Radiation Physics, Lund University, Lund, Sweden

**Keywords:** Survey, SGRT, Clinical practices, Surface-guided, Motion management

## Abstract

**Introduction:**

Surface Guided Radiation Therapy (SGRT) is being increasingly implemented into clinical practice across a number of techniques and irradiation-sites. This technology, which is provided by different vendors, can be used with most simulation- and delivery-systems. However, limited guidelines and the complexity of clinical settings have led to diverse patterns of operation. With the aim to understand current clinical practice a survey was designed focusing on specifics of the clinical implementation and usage.

**Materials and methods:**

A 32-question survey covered: type and number of systems, quality assurance (QA), clinical workflows, and identification of strengths/limitations. Respondents from different professional groups and countries were invited to participate. The survey was distributed internationally via ESTRO-membership, social media and vendors.

**Results:**

Of the 278 institutions responding, 172 had at least one SGRT-system and 136 use SGRT clinically. Implementation and QA were primarily based on the vendors’ recommendations and phantoms. SGRT was mainly implemented in breast RT (116/136), with strong but diverse representation of other sites. Many (58/135) reported at least partial elimination of skin-marks and a third (43/126) used open-masks. The most common imaging protocol reported included the combination of radiographic imaging with SGRT. Patient positioning (115/136), motion management (104/136) and DIBH (99/136) were the main applications.

Main barriers to broader application were cost, system integration issues and lack of demonstrated clinical value. A lack of guidelines in terms of QA of the system was highlighted.

**Conclusions:**

This overview of the SGRT status has the potential to support users, vendors and organisations in the development of practices, products and guidelines.

## Highlights

• First survey of SGRT clinical practice in Europe and beyond.
• Wide application range (sites and techniques) with diverse clinical use.
• Strong reliance on vendors for methods, training and quality assurance.
• Hurdles include cost, integration issues and lack of demonstrated clinical value.
• Despite some weaknesses a majority believe that SGRT will become standard of care.

## Introduction

Surface guided radiotherapy (SGRT) has been adopted into clinical practice in many radiotherapy institutions for patient setup, monitoring and gating [1], [2]. The main advantage of SGRT is to assess patient positioning in real-time without using ionising radiation [1], [2], [3], [4], [5]. The large field-of-view (FOV) for patient setup provides information about the patient’s anatomical topography [6] including rotations [7]. SGRT has been reported to either improve patient’s positioning [7], [8], [9], [10], [11], [12], [13], [14], [15], or provide comparable accuracy as 3-point localization [6], [9], [16] while improving efficiency[15], [17], [18], [19]. Due to increased information about the patient position and the standardised workflows, SGRT has the potential to greatly impact the quality and safety of radiation treatments [20]. Different installation settings are possible, as a result of several commercially available SGRT-systems, combined with most simulation and delivery systems.

Recognising the spread of this technology, the *European SocieTy for Radiotherapy and Oncology* (ESTRO) in collaboration with the *American Association of Physicists in Medicine* (AAPM) included a SGRT-topic in the “3rd Physics Workshop: Science in Development” to promote exchange between SGRT stakeholders and to support future recommendations for clinical applications and quality assurance (QA) of SGRT-systems [21]. One of the deliverables of the workshop was a survey conducted with the aim to provide insight into SGRT practices at radiotherapy institutions in Europe and internationally, with a focus on the prevalence, commissioning/implementation/QA, user-training, clinical workflows and user perceptions of SGRT’s strengths and limitations. As a second deliverable, ACROP-guidelines dedicated to SGRT clinical practice will be forthcoming.

## Material and methods

### Survey design and distribution

A 32-question survey was designed by SGRT-experienced physicists and radiation therapists (RTTs) with the aim of understanding current SGRT practices and prioritising guideline development. It was divided into four aspects: (i) prevalence of systems; (ii) acceptance, commissioning, and clinical implementation; (iii) clinical use; and (iv) perception of advantages and limitations. Depending on the user’s experience, as multiple choices and open answers were included, the completion time could vary between 5 and 30 min.

The target participants were current and potential users of this technology from different professional groups (RTTs, Physicists, Radiation Oncologists, and others). Distribution channels included ESTRO newsletter, vendors, social media and mailing-lists. The survey was available online between August and October 2020. The survey template is in the supplementary materials.

### Data processing and analysis

Answers were collected using an ESTRO SurveyMonkey (*Momentive Inc, USA*). The data was reviewed to improve data quality, i.e., to avoid duplicate, inconsistent, or contradictory answers within the same institution. Answers from non-existent names/cities/facilities were excluded. The difference between the start and end times of the survey was considered as a filtering parameter, e.g., <5 min for institutions with SGRT was considered not to be a realistic response. If there were multiple answers from the same person at the same clinic, the reasonable response with the latest timestamp was retained. If there were multiple answers from the same institution from different individuals, answers to each question were either concatenated (if similar or complementary) or ignored (if contradictory). The open text responses were converted into keywords, which were grouped into representative categories for statistical analysis. The attribution of keywords was cross-checked by 2–3 authors. After the first round of data processing, a script utilising the same criteria described above was used to verify the data used for analysis. This was performed in Excel (*Microsoft Corporation, USA*) by teams of two or more authors per section.

## Results

### Data distribution

**Fig. 1:**
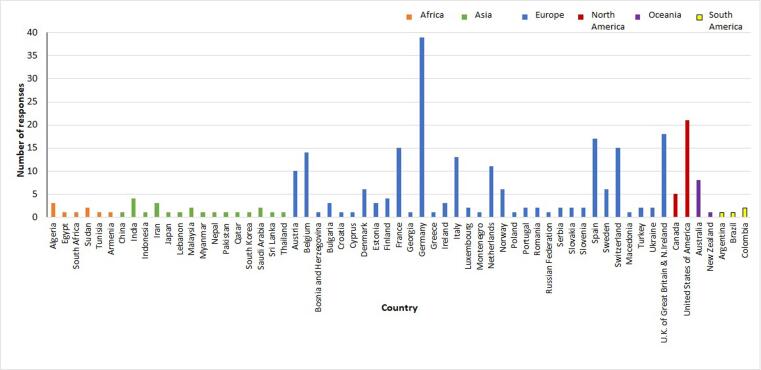
Distribution of the responders by country and continent. Note: the United Nations classification was used.

Of the total 294 responders, the majority (73.5%) were medical physicists (n = 216), followed by RTTs (16.3% (n = 48)), physicians (6% (n = 20)), researchers (2.0% (n = 6)) and others (1.4% (n = 4)). After processing, responses from 278 individual institutions spanning 62 countries, 207 European and 71 non-European, were available for analysis (Fig. 1).

### Prevalence of SGRT-systems

The vendors and recent publications [22] have encouraged the term “surface guided radiotherapy” to describe the integration of surface imaging into the radiotherapy workflow. The survey results indicate that most users have acclimated to the term with over 80% (n = 239) of respondents preferring the acronym “SGRT” over other options including “OSMS”, “SIGRT”, “SI”, and “OSM”.

Of the 278 responding institutions, 62% (n = 172) had at least one SGRT-system. Most installations were on traditional (C-arm) linear accelerators (linacs) from the main linac vendors: 59% (n = 113) *Varian Medical Systems* (Palo Alto, USA) or 29% (n = 56) *Elekta AB* (Stockholm, Sweden). Only 5.2% (n = 10) of SGRT-systems were installed on particle therapy machines and 6.7% (n = 13) were installed on either *Tomotherapy* (Accuray Inc, Sunnyvale, USA), Varian Halcyon, a *Siemens* Linac (Siemens Healthcare, Erlangen, Germany) or a *Cyberknife* (Accuray Inc, Sunnyvale, USA). In 44 institutions, SGRT-systems were also installed on computed tomography (CT) simulators.

From 172 responding institutions, which stated that they have at least one SGRT-system installed, 42% were equipped with only one SGRT-system, 34% with two systems, 19% of institutions reported using SGRT on three or more linacs, and 5% did not report the number of installed systems. Regarding the ratio of treatment machines with and without SGRT, 35% of institutions had SGRT installed on all treatment machines, while 69% had it installed on at least half of their linacs (see supplementary materials).

Among the vendors, *VisionRT/OSMS* (London, UK) was most prevalent, with 49% (n = 89), followed by *C-RAD* (Uppsala, Sweden) with 37% (n = 68), *Brainlab* (Munich, Germany) with 7% (n = 13), and *Varian Identify/HumediQ* with 7% (n = 12).

Of the 136 responding to the question regarding the duration of SGRT clinical use, 26% stated less than one year, 41% between 1 and 3 years and only 33% more than 3 years, which indicates a recent adoption of this technology.

### Acceptance, commissioning and clinical implementation

The survey inquired about the time spent by institutions on: (1) installation and acceptance (2) commissioning and (3) clinical implementation. Table 1 demonstrates that many institutions spent more than 48 h on each process.

**Table 1:** Distribution of time spent on various SGRT implementation stages across institutions.

Time	Implementation Stage		
	Technical installation + Acceptance testing (n = 117)	Commissioning (Testing and definition of QA-protocols) (n = 116)	Clinical implementation (Decision about processes, detailed description workflows, staff training) (n = 121)
<5 h	13%	19%	7%
5–10h	22%	24%	18%
10–24 h	14%	9%	16%
25–48 h	20%	11%	9%
>48 h	32%	36%	50%

Most respondents, 94% (n = 132), followed vendor suggestions for commissioning, clinical implementation, and QA at a minimum. Specifically, 42% (n = 59) of the respondents used two sources of recommendations from vendor, literature, or peer-to-peer consultation, while 19% (n = 27) used three or more sources.

#### Commissioning and QA

The frequency of various QA procedures is summarised in Table 2. A consensus (>50%) is only found for the daily isocenter verification and annual end-to-end testing.

**Table 2:** Highest frequency* with which each of the QA items are performed. (*as many of these QA tests are commonly performed at different periodicities, only the most frequent scheme was considered in this analysis).

Test-type	Periodicity			
	Daily or before each patient	Weekly/monthly	Yearly/after service-intervention	Never/not necessary
Isocentre Verification	79% (n = 106)	18% (n = 24)	1% (n = 2)	2% (n = 3)
Image Quality (FOV, Reference Acquisition)	37% (n = 47)	30% (n = 38)	18% (n = 23)	16% (n = 20)
Static Accuracy	44% (n = 56)	29% (n = 37)	18% (n = 23)	9% (n = 12)
Reproducibility of motion trace (Dynamic Accuracy)	18% (n = 22)	28% (n = 34)	26% (n = 32)	28% (n = 35)
End-to-end Test	11% (n = 14)	14% (n = 17)	54% (n = 66)	20% (n = 25)

About half of respondents (54% (n = 76)) exclusively used phantoms provided by their respective vendors for commissioning and QA. Eight percent (n = 12) reported not using the phantoms provided by the vendor, but exclusively used either third-party commercial phantoms (4% (n = 6)) or adapted or built in-house phantoms (4% (n = 6)). Many institutions used the vendor-provided phantom in combination with third party commercial phantoms (24% (34)) and/or with built in-house phantoms (20% (28)).

#### Clinical implementation & staff training

Clinical implementation of SGRT was predominantly led by physicists, either alone (56% (n = 81)), together with RTTs (27% (n = 40)) or in a team with RTTs and physicians (9% (n = 13)). In some institutions, either RTTs (3% (n = 4)) or a combination of various professionals (5% (n = 7)) were responsible for SGRT implementation.

The majority (73% (n = 93)) attended training provided by the vendor, of which 38% (n = 35) reported training only a key-users team. Within the institution, the training was performed by a team of interdisciplinary key-users (47% (n = 27)), or led by only one staff-group, such as physicists (43%), RTT (9%) or others (2%). Physicians were included in the training in 47% (n = 53) of institutions, but not in 42% (n = 47) with inconclusive answers given by 13% (n = 11).

At 51% (n = 58) of institutions, staff adapted easily to the system, but at other institutions either all (29% (n = 33)) or some (20% (n = 22)) had difficulties adjusting their clinical practices to use SGRT. The main reasons identified were the training process itself (23% (n = 15)) and adoption of new workflows (17% (n = 11)).

### Clinical use of SGRT

#### Common treatment sites

**Fig. 2:**
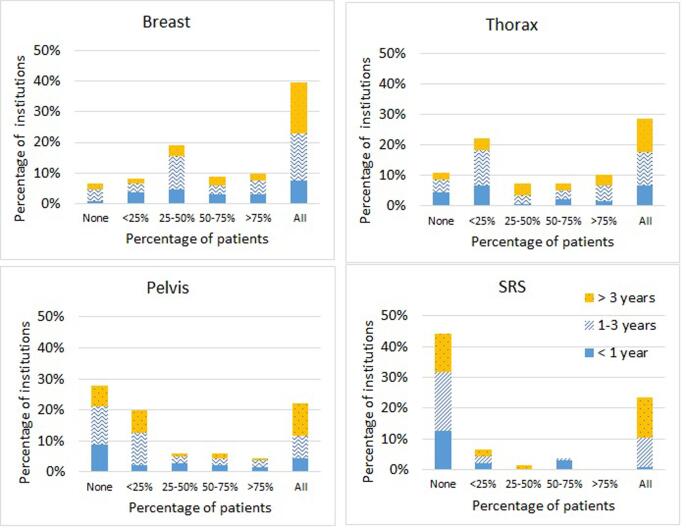
Histogram distribution of 136 institutions reporting on their use of SGRT for various treatment sites differentiated by the time since the SGRT implementation. Data for other treatment sites are available in the supplementary materials (Fig. A.1).

The most common application of SGRT was breast radiotherapy, in 85% of the 136 responding institutions, followed by thorax (76%), abdomen (65%), pelvis (58%), extremities (53%), head and neck (H&N) (32%) and other treatment sites (25%). Fig. 2 shows the frequency distribution of SGRT applications for some treatment sites with an indication of the SGRT experience by the respondents. New users (<1 year) tended to begin with breast RT, which is a well-established use of SGRT. Data for additional sites is in the supplementary materials.

#### Main SGRT-applications

**Fig. 3:**
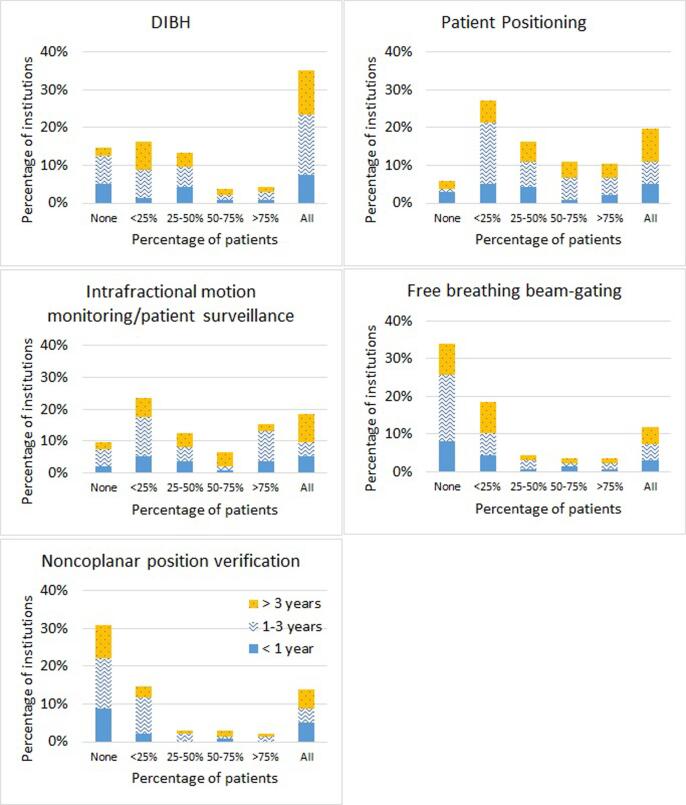
Histogram distribution of 136 institutions reporting on their use of SGRT for various clinical applications differentiated by the time since the SGRT implementation. Data is correspondant to all the treatment sites.

Over all treatment sites, the main applications of SGRT were reported to be patient positioning (85% (n = 115)), deep-inspiration breath-hold (DIBH) (73% (n = 99)), and intrafractional motion monitoring/patient surveillance (76% (n = 104)) (Fig. 3). Forty-two percent (n = 57) also reported its usage for free breathing beam-gating and 37% (n = 50) used it for noncoplanar position verification.

#### Modalities per treatment site

The reported frequency distributions of SGRT-applications revealed no considerable dependency on treatment sites for patient positioning or for intrafractional motion monitoring/patient surveillance (see supplementary materials). Both applications were reported to be used equally over all treatment sites by about 70% to 90% of the institutions. Application of SGRT for DIBH dominated in breast radiotherapy with 78% (n = 116). A smaller but non-negligible application of DIBH was reported for thorax (27% (n = 103)), SBRT (23% (n = 79)) and abdomen and pelvis (12% (n = 89)).

Other indications named for SGRT were: palliative radiotherapy, electron treatments, lung SBRT, DIBH for liver SBRT and for monitoring the swallowing motion of patients undergoing larynx irradiation.

#### Tattoos/Skin-Marks

The introduction of SGRT resulted in an overall elimination of tattoos/skin-marks at 23% (n = 31) of institutions. A partial elimination across all the treatment sites was reported by 20% (n = 27). Those who did not completely eliminate tattoos justified this by a limited number of SGRT-installations and/or treatment sites using SGRT-based workflows.

#### Open-Mask/Maskless

Furthermore, treatment with open masks in combination with SGRT was queried. Thirty-two percent (n = 43) affirmed this question with 51% of these institutions using it for SRS, 30% for whole brain irradiation, 28% for H&N, 12% for claustrophobic patients, 7% for either glioblastoma or larynx irradiation, 5% each for palliative patients and for intracranial RT and 2% for facial tumours. In addition, maskless treatment with SGRT was mentioned for H&N and whole brain irradiation by two respondents.

#### Complementary IGRT

**Fig. 4:**
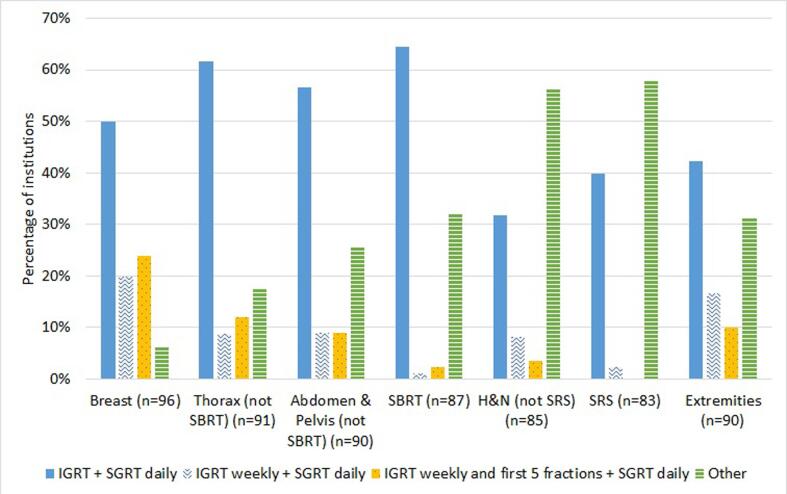
Distribution of different setup verification protocols per treatment sites. The most common combinations of IGRT and SGRT protocol were given as option, as well a “other” alternative with a open text field.

Institutions using SGRT for patient positioning were asked whether additional image-guided radiation therapy (IGRT) was used. The combination of daily IGRT and SGRT was reported as the most used protocol for patient positioning for the majority of treatment sites and ranged from 42% for extremities to 64% for SBRT (Fig. 4).

#### Reference surface

**Fig. 5:**
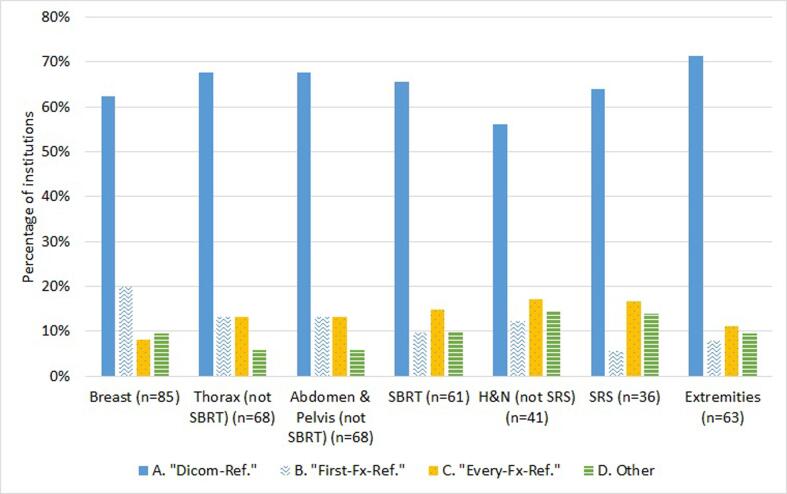
Distribution of reference surface used for patient positioning of various treatment sites. “Dicom-Ref.” refers to the surface obtained from the treatment planning system, “First-Fx-Ref.” to a surface acquired by the SGRT-system at the first treatment session and used in the following session, and “Every-Fx-Ref.” to a daily acquisition of a new SGRT-reference. Additionally, to these three options, the responders had the alternative to explain other imaging scheme (“Others”).

The digital imaging and communication in medicine (DICOM) surface from the treatment planning CT (DICOM-Ref.) was used as the reference surface for daily patient setup by the majority of respondents ranging from 56% to 71% for all treatment sites. Other reference surfaces, such as an SGRT camera-acquired surface at first fraction (First-Fx-Ref.), at each fraction (Every-Fx-Ref.) or other schemes, are infrequently used (6–20%) (see Fig. 5).

#### Treatment tolerances

**Fig. 6:**
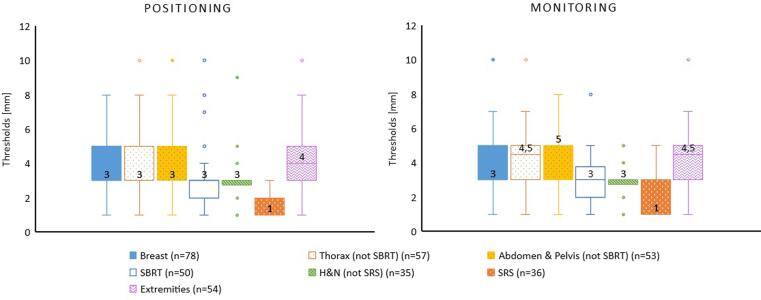
Reported thresholds used for initial patient positioning and intrafraction monitoring. Median values are displayed over each box plot and also indicated by a line. The results are displayed in function of the treatment site.

The reported distribution of thresholds per treatment site and radiation technique is shown in Fig. 6. Of 363 answers across treatment sites, the majority (75%) use the same thresholds for patient positioning and monitoring. The median threshold was 3 mm for breast, SBRT and H&N. Smaller thresholds (1 mm) were reported for SRS for positioning and monitoring. Slight differences in the median threshold in positioning and monitoring were reported for thorax, abdomen and pelvis as well as for extremities.

#### Patient-feedback-system

The use of visual feedback for breath-hold and free-breathing gating was assessed. Of the 105 respondents, 72% perform motion management with SGRT for which visual feedback is preferred, with 63% in breath-hold and 10% in free-breathing techniques, respectively.

### Perception of advantages and limitations of SGRT

When asked about the main limitations of extending SGRT to additional body sites, 98 institutions responded. These limitations can be summarized in a total of 110 keywords, and include: the presence of masks, clothes, and immobilisation devices (n = 27), staff acceptance (n = 18) and no redundant linac with SGRT-system (n = 16)).

**Fig. 7:**
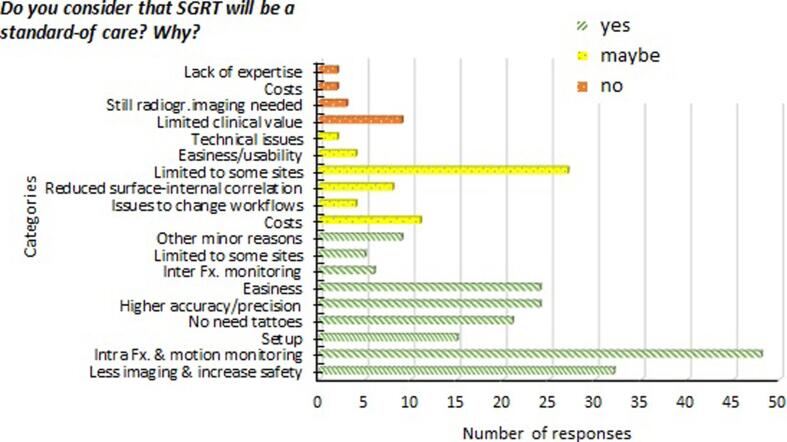
Distribution of reasons given to the question “Do you consider that SGRT will be the standard of care?” for the responders that justified their choice of ‘Yes’, ‘Maybe’, and ‘No’. More than one reason could be given by responder.

The question of whether SGRT could soon become the standard-of-care was answered by 223 institutions. Of those who agreed (56%), 39% cited the ability to manage and monitor intra-fraction motion. Of those who disagreed (9%), the main explanation cited was the lack of demonstrated clinical value. Of those who were undecided (35%), 57% indicated that there are limited clinical indications for SGRT. Further details can be found in Fig. 7.

Respondents were asked to rank the factors that presented barriers to SGRT implementation, where 1 was the most important. Of the 200 institutions answering this question 65% ranked cost as the most important factor. Opinions were split regarding the second most important factor and included: time constraints for implementation (24%), lack of understanding (24%), limited staff (16%), cost (16%) and staff training (14%).

The question of whether SGRT is financially covered by insurance across the institutions was difficult to address. Although most respondents (69%) stated that no extra reimbursement for SGRT is available, reimbursement is a complex field and can vary not just among countries and jurisdictions but also among individual clinics.

The main challenges for SGRT in the next 5 years were addressed by 136 respondents. Of 197 answers, the main challenges reported were system performance and integration (33%), cost/limited-number of systems (20%), and lack of guidelines and workflow-optimisation issues (19%).

Finally, respondents were asked which aspects should be prioritized by the ESTRO working group. Of128 responders, a total of 186 answers were collected. Twenty-six percent highlighted that the QA process needs to be addressed, 22% suggested the development of clinical guidelines and consideration of quality manuals, while 13% pointed out the need for workflows (including markerless treatments) and tolerances. Other aspects such as clinical value (10%), technical characteristics (10%), staff training (9%), requirements/drawbacks (4%), correlation surface-internal (3%), costs (2%) and special techniques (2%) were mentioned.

## Discussion

This survey assessed the prevalence of SGRT-systems in and outside of Europe with a focus on the different strategies for commissioning, quality assurance and clinical implementation. It provides an overview of current clinical practices, and insights into perceived advantages and limitations by current and prospective users.

In the absence of dedicated guidelines, clinical implementation has relied on vendor recommendations, published literature, and collaboration between institutions. This placed great responsibility on individual institutions to ensure safe clinical implementation and in the vendor-led or staff-led training program. It is worth noting that according to the survey-data (Section 3.3) almost half of the staff still encountered challenges adopting SGRT. This is possibly due to difficulties in transitioning from using traditional data (i.e., 3-point setup) to more complex data provided by SGRT-systems [21]. Also, the fact that training and implementation is mainly led by medical physicists may be a barrier to other staff groups and might thereby limit maximising the potential of SGRT.

Important data captured from the survey regarding the users’ experience include the need to allocate an adequate number of hours (>40 h) for the installation/acceptance and commissioning as well as for clinical implementation. An implementation timeline similar to that of other IGRT modalities was suggested [23].

Concerning clinical SGRT-workflows, breast is still the most common application site, as confirmed by the number of publications [4]. Reasons include: the clinical advantage compared to laser positioning [14], good surface-to-target correlation [24], and the availability of a large number of publications, which have served as guidelines for users initiating SGRT. Unlike other sites, for which daily x-ray based IGRT remains the gold standard [5], [8], breast RT does not typically employ daily x-ray imaging. Thus, only for this site more than 25% of institutions use SGRT as the sole IGRT modality for daily positioning verification.

Another important decision when establishing clinical protocols is the choice of the reference-surface (DICOM or camera-acquired). Although many vendors emphasize the technical advantage of a camera-acquired reference, the use of a DICOM reference provides more confidence to the users because it matches the CT simulation position [2], [20]. Respondents indicated that it was the predominant reference surface used in all the treatment sites.

Regarding the thresholds, no significant differences were observed between patient positioning and patient monitoring, which could be explained by a workflow mainly recommended by vendors (39%) or radiation oncologists (37%). The median action limit was around 3 mm for all the cases with the exception of SRS treatments (1 mm), which is comparable to values used for IGRT protocols. However, in all the cases a wide range of thresholds among users was observed, which points to a lack of consensus guidelines and/or different protocols (e.g. different reference surfaces, different treatment machines and SGRT-systems combinations, use of tattoos, daily IGRT protocols, etc.).

Although several published works about the elimination of tattoos exist [18], only 25% did so, with the main reason being the limited number of installed systems, which corresponds to 35% of institutions that have SGRT at all their machines. Therefore, backup strategies to allow patient transfer between machines would need to be implemented, as definition of alternative workflows and/or maintenance of skin-marks.

The extension of SGRT to all body regions is still reported as a challenge due to the need to keep the patient’s skin uncovered during treatment (patient comfort), but also due to staff or equipment shortages (40%). Nevertheless, the majority of responders (56%) believe that SGRT has the potential to become a new standard-of-care since it could address pre-existing workflow challenges. This is in agreement with a recent publication addressing future developments in SGRT [21].

The main concerns regarding SGRT use include the system integration and performance, costs or future costs for additional systems, and the absence of guidelines and optimization protocols. Other concerns include extending SGRT to other sites, lack of demonstrated clinical value, and staff training. As conflicts in system integration and performance were the major challenges seen by the survey participants, SGRT vendors should make continuous effort to improve these aspects of their systems.

Some limitations in the survey are recognised. First, although the response rate was in line with other similar surveys [22], it was limited to 300 institutions and the geographical distribution was not uniform across countries. Because the survey was mainly designed and distributed by physicists, this could have resulted in an overrepresentation of their viewpoint compared to other radiation oncology professionals. Also, including vendors in the distribution channel of the survey may have induced some bias. Considering the fast adoption of this technology in recent years [22], the increase of vendors entering this market, and the growing availability of SGRT on various treatment machines, SGRT practices might change quickly in the near future. Nevertheless, this survey did provide valuable insight into SGRT implementation in 2020 and some of the challenges encountered during this process.

## Conclusions

This work provides an overview of the status of SGRT in the Radiation Oncology community in Europe and beyond in terms of its prevalence, technical and clinical implementation. While SGRT is becoming part of clinical practice, its implementation has been challenging due to lack of guidelines and resources (i.e., time, staff, money) particularly since the clinical value of SGRT has not been unequivocally established and its clinical use is typically not reimbursed [21].

Given that this non-ionizing imaging modality can be used to increase treatment safety and quality via intra-fractional monitoring and the support of DIBH, as well as increase efficiency via the support of patient setup [20], institutions would benefit from consensus guidelines on SGRT to expedite its safe adoption.

The presented results can serve as an orientation for current and prospective users. Issues to consider when planning and negotiating their SGRT implementation or expansion are highlighted. Professional organisations can benefit when strategizing their future efforts. Vendors should be guided by them when developing their products and education portfolios.

## Declaration of Competing Interest

The authors declare that they have no known competing financial interests or personal relationships that could have appeared to influence the work reported in this paper.
