# Supplementary material for: Surface guided radiation therapy: An international survey on current clinical practice
Source: Tech Innov Patient Support Radiat Oncol. 2022 Mar 30;22:1–8. doi: 10.1016/j.tipsro.2022.03.003 (PMC8984757; doi:10.1016/j.tipsro.2022.03.003)
Supplement: Supplementary data 1 [file mmc1.pdf]

Supplementary material

A) Additional Plots

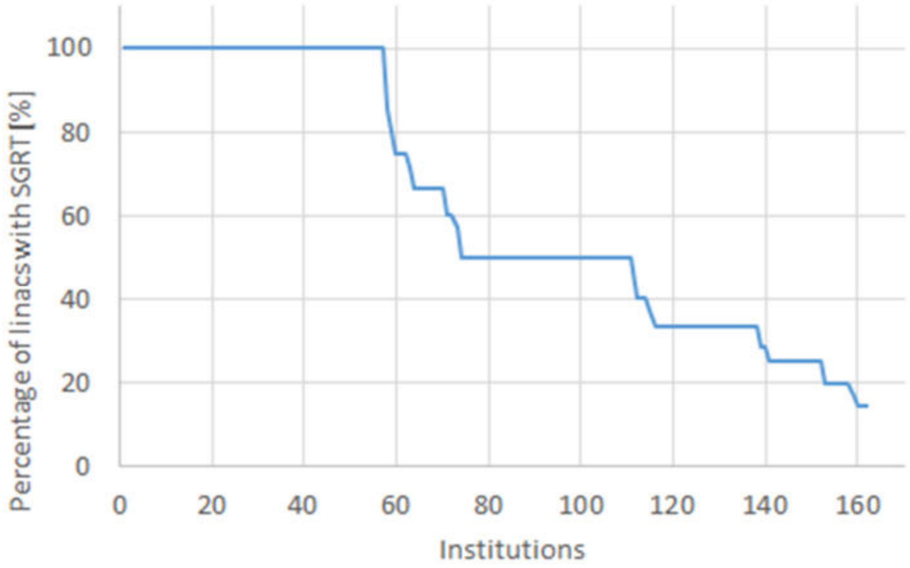

Figure A1: Number of SGRT equipped linacs relative to total number of linacs in responding institutions

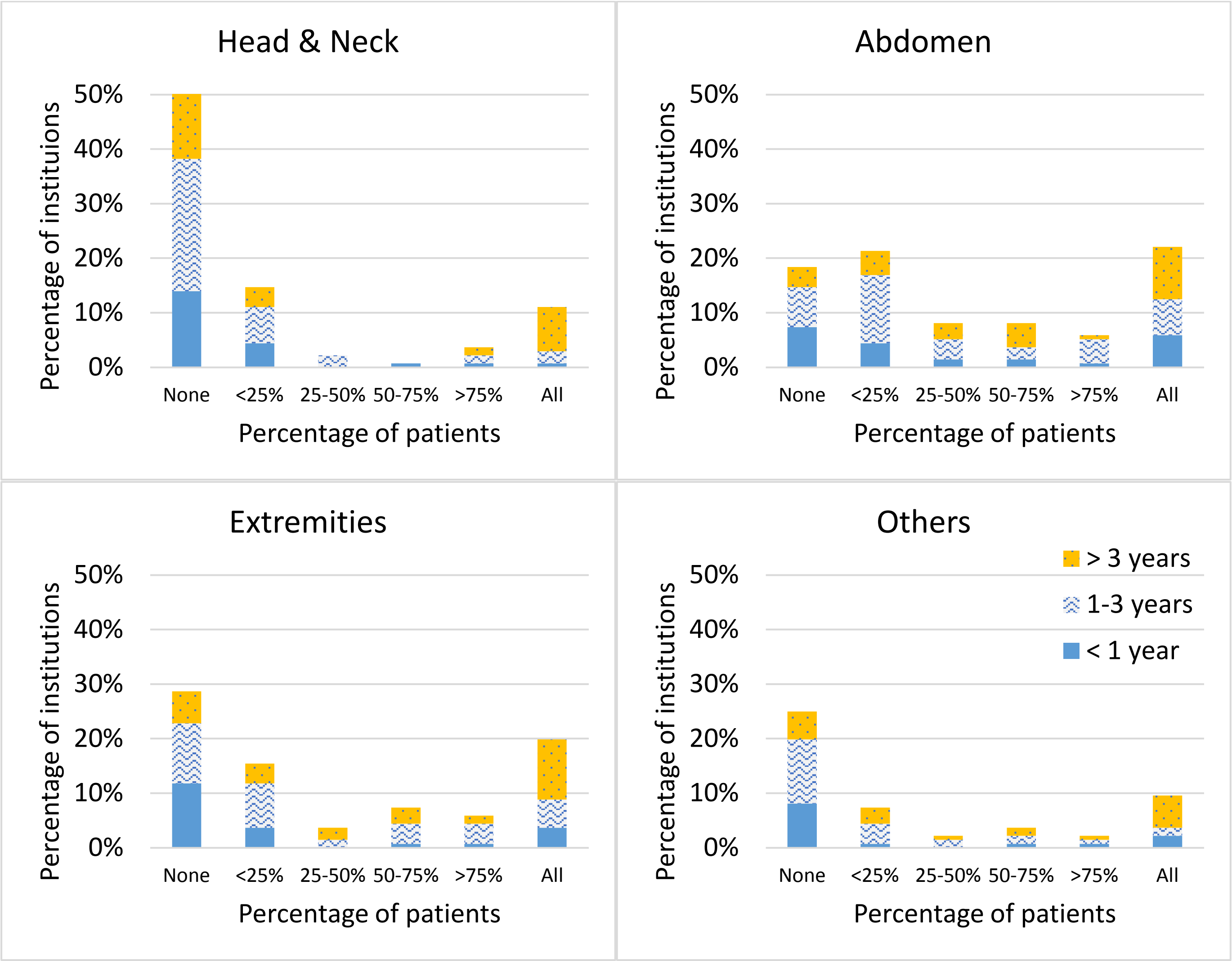

Figure A2: *Relative number of institutions reporting the frequency distribution of SGRT applications for various treatment sites differentiated by the time period of SGRT clinical use.*

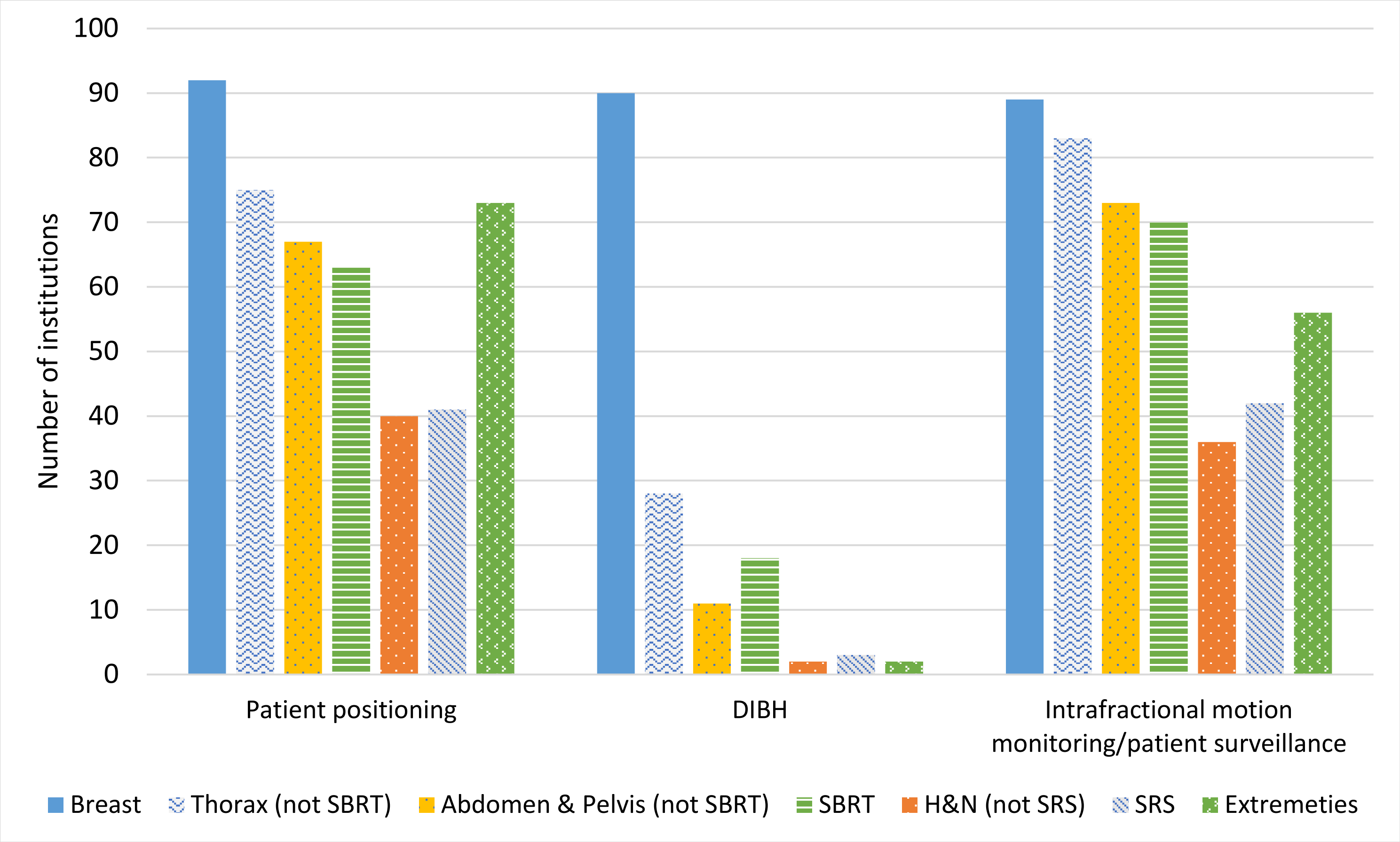

***Figure A3**: Reported SGRT-applications per treatment site. The combination of different applications per treatment site is possible.*

B) <u>Distributed Survey</u>

Surface Guided Radiation Therapy - Current Status

1. Surface Guided Radiation Therapy - Current Status

During the 3rd ESTRO physics workshop, a group of SGRT users with various experiences came together to discuss the clinical use and future of this technology. Given the lack of specific recommendations on SGRT to date, we discussed various approaches to QA and commissioning, and the establishment of clinical workflows. One of the outcomes of the workshop was the creation of an ESTRO Working Group dedicated to Surface Guided RT.

This survey was designed with the aim to understand the current SGRT-practices, adapt recommendations to the European users, and prioritize guidelines-needs.

Note: The survey may be extensive for some users (10-25min). You can opt to skip some questions or only shortly explain your answers. However, your feedback is very important and will allow us to elaborate adequated guidelines to your needs.

We welcome Surface-Guided-RT users and non-users (but who intent to install Surface-Guided) to fill this survey. Both Physicists, RTTs, and other professionals involved with this technology are invited to taking part.

Thank you for participating in our survey.

Surface Guided Radiation Therapy - Current Status

2. Section I : Facility Characterization

* 1. Please provide your data.

Note: Data will be used in anonymized way. Institution Name collected only to avoid duplication, not to be published

Name

Institution Name

City/Town

Email Address

* 2. Specify your Role.

* 3. Specify your facility country:

* 4. Which acronym do you use when referring to Surface guided radiation therapy?

- [ ] SI
- [ ] SIGRT
- [ ] SGRT
- [ ] OSM
- [ ] OSMS
- [ ] Other (please specify)

* 5. Do you have Surface-Guided-RT installed at your Clinic?

- ( ) Yes
- ( ) No

3. Section I : Facility Characterization

* 6. On which machine(s) do you have surface guided installed/under-installation?

- [ ] Elekta
- [ ] Varian (Truebeam, C-series,..)
- [ ] Varian Halcyon
- [ ] Siemens
- [ ] Tomotherapy
- [ ] Other (please specify)
- [ ] Cyberknife
- [ ] CT scanner
- [ ] Particle therapy fixed beam line
- [ ] Particle therapy gantry

* 7. Specify the number of Linacs with/without a system.

	# Linacs
Without	
Under installation/acceptance/commissioning	
Yes, in clinical use	

Other (please specify)

* 8. Which SGRT Vendor(s) do you have installed/under-installation in your clinic?
Can you please specify which products do you have?

- [ ] BrainLab
- [ ] C-RAD
- [ ] Varian Identify / Humediq
- [ ] Vision RT / OSMS
- [ ] Other Vendor

Please specify the installed (or under installation) products

4. Section II: Commissioning, QA and Implementation

9. Which staff was responsible for the implementation of the system?

- ☐ Physicist
- ☐ Physician
- ☐ RTT
- ☐ Administration
- ☐ Other (please specify)

10. Define the periodicity of your QA for the following parameters:

	Daily	Weekly / Monthly	Yearly / After Service-Intervention	Patient-Specific/before each patient	Never / not necessary
Isocenter	☐	☐	☐	☐	☐
Image Quality (FOV, Reference Acquisition)	☐	☐	☐	☐	☐
Static Accuracy	☐	☐	☐	☐	☐
Reproducibility of motion trace (Dynamic Accuracy)	☐	☐	☐	☐	☐
End-to-end Test	☐	☐	☐	☐	☐
Other	☐	☐	☐	☐	☐

Other (please specify)

11. Which phantom(s) did/do you use for SGRT system commissioning and ongoing QA? <u>And please specify the product name.</u>

- ☐ Phantoms provided by SGRT vendor
- ☐ Adapted or in-house built phantoms
- ☐ Third party commercial phantoms

Please specify

12. Which recommendations for implementation, commissioning and QA of SGRT did you follow? (please specify)

- ☐ Vendors suggestions
- ☐ Peer-to-peer consultation
- ☐ Literature guidelines
- ☐ Others

Specify your source

13. How many hours has your department spent on the implementation of the first SGRT system in your institution? I.e. time between the installation and the first patient?

	<5hrs	5-10hr	10-24hrs	25-48hrs	>48hrs
Installation + Acceptance (with vendor)	○	○	○	○	○
Commissioning	○	○	○	○	○
Definition and documentation workflows	○	○	○	○	○
(Initial) Staff training	○	○	○	○	○

Other (please specify)

14. Staff training: Who performed the training? Comment any relevant aspect about the training process.

Who performed the training?

Major Issue?

Staff accepted the system easily?

Did you include clinicians in the training process?

Other comments?

* 15. Are you already using the system clinically?

- Yes, less than one year
- Yes, between 1-3 years
- Yes, more than 3 years
- No, under installation/commissioning

5. Section III: Clinical Workflows

The next questions concern to your workflow for each body site (please skip the cases where you don’t use Surface-guided)

* 16. Which **percentage of patients** treated at the following sites do you use surface guidance for? (provide a percentage 0-100% for each)

	None	<25%	25-50%	50-75%	>75%	All	NA/Don't know
Breast	◯	◯	◯	◯	◯	◯	◯
Thorax	◯	◯	◯	◯	◯	◯	◯
Abdomen	◯	◯	◯	◯	◯	◯	◯
Pelvic	◯	◯	◯	◯	◯	◯	◯
Head & Neck (not SRS)	◯	◯	◯	◯	◯	◯	◯
SRS	◯	◯	◯	◯	◯	◯	◯
Extremities	◯	◯	◯	◯	◯	◯	◯
Others	◯	◯	◯	◯	◯	◯	◯

17. With which intent is Surface-guidance in your department applied to the following patients:

	Patient positioning	DIBH	Free breathing beam-gating	Intrafractional motion monitoring/patient surveillance	NA/Don't know
Breast	☐	☐	☐	☐	☐
Thorax (not SBRT)	☐	☐	☐	☐	☐
Abdomen & Pelvic (not SBRT)	☐	☐	☐	☐	☐
SBRT	☐	☐	☐	☐	☐
Head&Neck (not SRS)	☐	☐	☐	☐	☐
SRS	☐	☐	☐	☐	☐
Extremities	☐	☐	☐	☐	☐

Other (please specify)

* 18. Which **percentage of patients** do you use surface guidance for the following applications? (provide a percentage 0-100% for each)

	None	<25%	25-50%	50-75%	>75%	All	NA/Don't know
Patient positioning	○	○	○	○	○	○	○
DIBH	○	○	○	○	○	○	○
Free breathing beam-gating	○	○	○	○	○	○	○
Intrafractional motion monitoring/patient surveillance	○	○	○	○	○	○	○
Noncoplanar position verification	○	○	○	○	○	○	○

19. Has the use of SGRT led you to eliminate tattoos/skin marks for patients treated on the treatment machines equipped with SGRT technology?

○ Yes

○ No

○ Other (e.g. only for some locations. Please specify.)

20. Do you treat any patients with open masks and surface guidance?

○ No

○ Yes (Please specify details, including body-sites)

21. To obtain a more detailed overview of the current practices, we would like to get additional information about your workflows.
Are you willing to answer five additional questions in this section?

○ Yes

○ No

Surface Guided Radiation Therapy - Current Status

6. Section III: Clinical Workflows - Detailed

The next questions concern to your workflow for each body site (please skip the cases where you don’t use Surface-guided)

22. Which were/are the main limitations to extend SGRT to more body-sites?

23. (if SGRT used for positioning in your department)
Do you use additional image guidance in combination with SGRT for positioning? If so, which protocol do you follow?

	IGRT + SGRT daily	IGRT weekly + SGRT daily	IGRT weekly and first 5 fractions + SGRT daily	NA/Don't know
Breast	○	○	○	○
Thorax (not SBRT)	○	○	○	○
Abdomen & Pelvic (not SBRT)	○	○	○	○
SBRT	○	○	○	○
Head & Neck (not SRS)	○	○	○	○
SRS	○	○	○	○
Extremities	○	○	○	○

Other (please specify)

24. (if SGRT used for positioning in your department)
Which reference do you use for daily patient setup?

	Reference
Breast	
Thorax (not SBRT)	
Abdomen & Pelvic (not SBRT)	
SBRT	
Head & Neck (not SRS)	
SRS	
Extremities	

Other (please specify)

25. Specify the threshold values for each body site.

	Do you use different values for positioning and monitoring?	Monitoring	Positioning	On the basis of what did you specify the values?
Breast				
Thorax (not SBRT)				
Abdomen & Pelvic (not SBRT)				
SBRT				
Head & Neck (not SBRT)				
SRS				
Extremities				

Other (please specify)

26. Do you use visual feedback devices for breath-hold and gating?

- [ ] We don't perform motion management with SGRT (neither breath-hold or free-breathing gating)
- [ ] Yes, for free-breathing gating
- [ ] Yes, for breath-hold patients
- [ ] No (why?)
- [ ] Please comment your choice

7. Section IV: Additional Information

27. Would you consider SGRT to be soon the standard of care and that all linacs should be equipped with SGRT technology? Please explan your opinion.

- Yes
- No
- Maybe

Please shortly explain your opinion

28. Does your clinic consider the acquisition of a (new) SGRT in the next 2 years?

- Yes
- No
- I don't know

29. Please rank the following factors in order of which they present(ed) hurdles to implement SGRT in your clinic: (where 1= most important)

Costs

☐ N/A

No time for a installation/commissioning/workflow-adaptation

☐ N/A

Internal policies

☐ N/A

Staff Training

☐ N/A

Limited Staff involved into the implementation process

☐ N/A

Lack of clinic understanding why SGRT can be beneficial

☐ N/A

Other

☐ N/A

30. Which are the main challenges for SGRT in the next 5 years?

31. Which questions should the Surface-guided ESTRO working-group consider as priority?

32. Is SGRT application financially covered by insurances (or there is an extra reimbursement) in your institution?

- ◯ Yes
- ◯ No
- ◯ Sometimes (please, specify)
